# Visualization of neuritic plaques in Alzheimer’s disease by polarization-sensitive optical coherence microscopy

**DOI:** 10.1038/srep43477

**Published:** 2017-03-06

**Authors:** Bernhard Baumann, Adelheid Woehrer, Gerda Ricken, Marco Augustin, Christian Mitter, Michael Pircher, Gabor G. Kovacs, Christoph K. Hitzenberger

**Affiliations:** 1 Medical University of Vienna, Center for Medical Physics and Biomedical Engineering, A-1090, Vienna, Austria; 2 General Hospital and Medical University of Vienna, Institute of Neurology, A-1090, Vienna, Austria; 3 General Hospital and Medical University of Vienna, Department of Biomedical Imaging and Image-guided Therapy, A-1090, Vienna, Austria

## Abstract

One major hallmark of Alzheimer’s disease (AD) and cerebral amyloid angiopathy (CAA) is the deposition of extracellular senile plaques and vessel wall deposits composed of amyloid-beta (A*β*). In AD, degeneration of neurons is preceded by the formation of A*β* plaques, which show different morphological forms. Most of them are birefringent owing to the parallel arrangement of amyloid fibrils. Here, we present polarization sensitive optical coherence microscopy (PS-OCM) for imaging mature neuritic A*β* plaques based on their birefringent properties. Formalin-fixed, post-mortem brain samples of advanced stage AD patients were investigated. In several cortical brain regions, neuritic A*β* plaques were successfully visualized in tomographic and three-dimensional (3D) images. Cortical grey matter appeared polarization preserving, whereas neuritic plaques caused increased phase retardation. Consistent with the results from PS-OCM imaging, the 3D structure of senile A*β* plaques was computationally modelled for different illumination settings and plaque sizes. Furthermore, the birefringent properties of cortical and meningeal vessel walls in CAA were investigated in selected samples. Significantly increased birefringence was found in smaller vessels. Overall, these results provide evidence that PS-OCM is able to assess amyloidosis based on intrinsic birefringent properties.

More than 46 million patients suffered from Alzheimer’s disease (AD) in 2015, making it the most common form of dementia in the world^1^. With a prevalence forecasted to double every 20 years^2^ and ageing populations worldwide, AD is not only growing with epidemic prevalence but is also posing an increasing social and financial burden^3^,^4^,^5^. In order to tackle the disease at a very early stage, the pathogenesis of AD needs more investigation. Hence, there is a growing demand for the development of new methods for both diagnosis and treatment of AD.

The diagnosis of AD is challenging. Clinically, AD manifests with progressive cognitive impairment with increasingly accompanying neurocognitive and neurological disturbances^6^. AD is characterized by degeneration of neurons in the brain preceded by the formation of extracellular senile plaques composed of amyloid-beta (A*β*) protein and intracellular neurofibrillary tangles composed of hyperphosphorylated tau protein^7^. Based on biomarkers detecting A*β*, the concept of preclinical AD has been developed^8^. This concept implements that preventive or disease modifying therapies could be already initiated in an early phase of the disease. Currently, reliable – i.e. definite – diagnosis of AD requires (post-mortem) histological analysis of central nervous system (CNS) tissue in order to confirm these pathological changes^9^. Clinical (pre-mortem) diagnosis relies on the assessment of cognitive impairment and memory, and is prone to inaccuracy due to the similarity of symptoms with other diseases such as depression^10^. Significant efforts have been made to identify objective methods as well as reliable and early biomarkers for AD, in particular since chances of effective therapy decrease once the clinical symptoms have already appeared. Being able to detect brain atrophy and cortical A*β* aggregates, respectively, magnetic resonance imaging (MRI) and positron emission tomography (PET) are promising imaging techniques^11^,^12^,^13^,^14^. Other studies have shown that AD patients have higher levels of tau and lower levels of A*β*42 in their cerebrospinal fluid^15^. However, to date, most of the sophisticated methods for AD diagnosis have not yet been accurate enough, are costly, and access is limited to certain patient groups – hence the search for new methodological approaches continues.

The gold standard for the diagnosis of AD is the neuropathological examination of post-mortem brain including screening for A*β* and tau protein deposits using immunohistochemical methods. Extracellular A*β* deposits show different morphologies from perineuronal and diffuse deposits to so-called focal deposits, which may be associated or not to a neuritic corona^16^. In mature plaques, A*β* has a fibrillary substructure and exhibits birefringence (Fig. 1(A,B))^17^,^18^,^19^. The intrinsic birefringence of A*β* can be increased by staining with Congo red, a dye commonly used as a laboratory aid for the diagnosis of amyloidosis^20^,^21^. A*β* plaques and vessels affected by amyloidosis (as in cerebral amyloid angiopathy) appear reddish in bright field micrographs after staining with Congo red and have a positive signal in polarization contrast micrographs^17^, which is often referred to as “apple green birefringence”^18^,^22^.

Optical coherence tomography (OCT) is a noninvasive modality for three-dimensional (3D) imaging of transparent and translucent samples and tissues^23^. Mostly applied in ophthalmology, OCT provides 3D images of tissue structures with micrometer scale resolution and rapid imaging speeds on the order of a few seconds per volume^24^. However, OCT has also been used for 3D imaging of brain tissue. Since tissue scattering properties depend on the internal microstructure, OCT has been demonstrated for contrasting lesions such as tumors in *ex vivo* human brain tissue^25^,^26^,^27^,^28^. In brain tissue of rodents, OCT based microscopy – optical coherence microscopy (OCM) – was successfully used for visualizing the cortical microstructure such as single neuronal cell bodies and myelin sheets *in vivo* and *in vitro*^29^,^30^. OCT angiography was able to visualize vascular networks in the brains of healthy animals as well as in tumor and stroke models^31^,^32^,^33^. Cerebral blood flow was furthermore assessed quantitatively with a Doppler OCT-based method^34^,^35^. In mouse models of AD, structural changes in the brain due to amyloidosis were imaged using OCM with dark-field illumination^36^. Cerebral tissues of human AD patients have – to the best of our knowledge – not yet been investigated with OCT based methods.

In this article, we present cerebral imaging of AD-related pathology using polarization-sensitive (PS) OCT. Other than standard OCT, which acquires images solely based on the intensity of light backscattered from tissue, PS-OCT can simultaneously measure its polarization state^37^,^38^,^39^. Since different biological structures affect the polarization of light differently, PS-OCT provides access to intrinsic, tissue-specific image contrast as well as to quantitative imaging^40^. In the brain, PS-OCT enables distinguishing grey from white matter^41^,^42^, and was demonstrated for noninvasive tractographic imaging of white matter fiber tracts in rat brains^43^,^44^. In the following, we demonstrate *ex vivo* PS-OCT imaging of neuritic A*β* plaques in the brains of AD patients. Complementary to the 3D image data, we used electron microscopy data to develop a computational model for polarization propagation in cortical A*β* plaques. Furthermore, we investigated the birefringent properties of cerebral vasculature in cerebral amyloid angiopathy (CAA) and controls.

## Methods

### Brain samples

Brain samples of patients diagnosed with end-stage AD (Braak stage V or IV, CERAD plaque score C, Thal phase 5) as well as with confirmed extensive CAA (Thal stage 3) were retrieved from the Neurobiobank of the Medical University of Vienna. Post-mortem specimens were obtained from patients who underwent autopsy at Medical University of Vienna. Informed consent for the use of their biological materials for research purposes was obtained by all patients. All methods were carried out in accordance with relevant guidelines and regulations. This study was approved by the institutional review board at Medical University of Vienna (approval number 396–2011).

From each post-mortem brain, several representative regions (i.e., mediotemporal/hippocampal, frontal, temporal, and occipital lobes) were sampled. Formalin-fixed brain tissues were directly imaged using PS-OCT prior to paraffin-embedding for histopathologic work-up. Paraffin blocks were cut at a thickness of 3 *μ*m for immunostaining. In addition, unstained sections were cut at a thickness of 50 *μ*m and deparaffinized for examination by PS-OCM.

### Histologic analysis

Histologic assessment was based on conventional Hematoxylin Eosin and Congo red stainings as well as immunohistochemistry for beta A4-amyloid (1:400, clone 6 F/3D, Dako, Glostrup, Denmark). Slides were assessed using a Nikon Eclipse 80i microscope (camera: Jenoptik ProgRes C5) comprising bright field and polarization contrast microscopy. For electron microscopy small areas of temporal cortex were postfixed in 1% osmium tetroxide for 1–2 hours, dehydrated through a series of graded ethanols and propylene oxide, and then embedded in Embed 812 resin. Ultrathin sections were stained with lead citrate and uranyl acetate. Specimens were examined using a transmission electron microscope (Carl Zeiss EM 109).

### PS-OCT apparatus and imaging protocol

**Figure 1:**
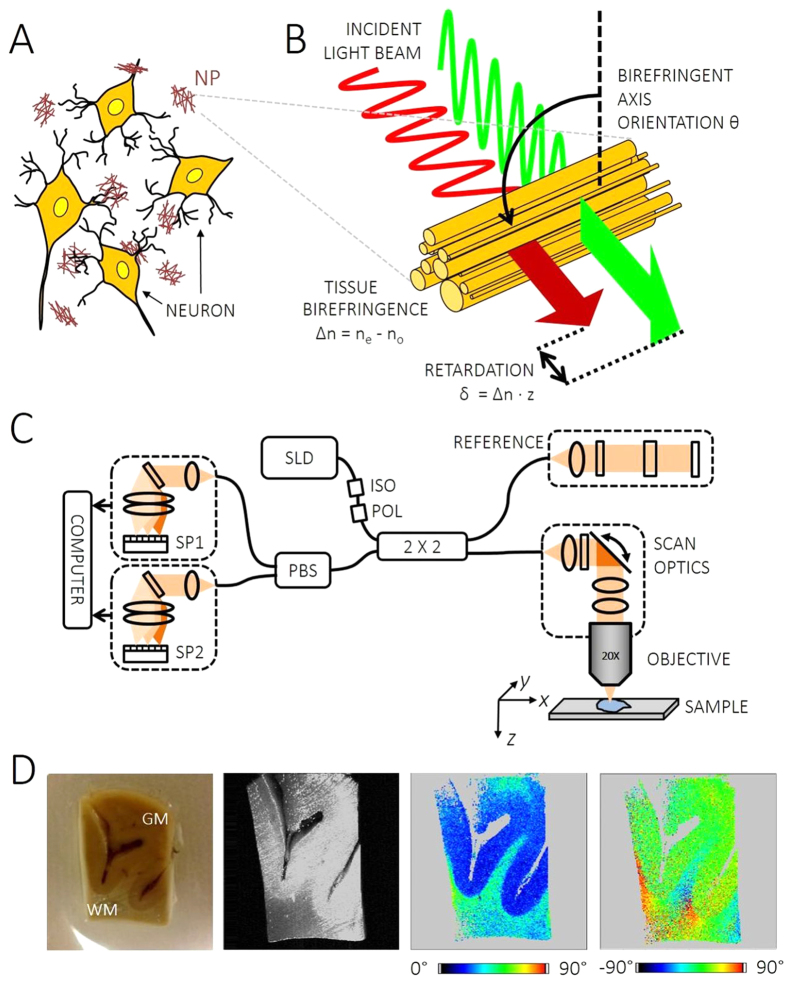
A*β* plaque birefringence and PS-OCT. (**A**) Neuritic plaques (NP) are extracellular deposits of A*β* around cortical neurons. Their fibrous substructure causes birefringence. (**B**) Birefringent fibers produce a phase retardation between orthogonally polarized light components. (**C**) Sketch of spectral domain PS-OCT device. The sample arm incorporates scan optics and an objective. The OCT signal is detected by two identical spectrometers (SP), one each for an orthogonal polarization state. SLD superluminescent diode, ISO optical isolator, POL polarizer, 2 × 2 fiber coupler, PBS polarizing beam splitter. (**D**) Photo (left, GM: grey matter, WM: white matter) and exemplary PS-OCT images of a formalin-fixed brain sample. The sample structure can be observed in the reflectivity image. Birefringent WM and polarization preserving GM can be distinguished in the phase retardation image extracted 50 *μ*m below the tissue surface in greenish and blue hue, respectively. Different fiber orientations give rise to rainbow-like color changes in the axis orientation image.

A PS-OCT prototype instrument presented earlier was slightly modified for imaging of brain samples^45^. In brief, the system was based on spectral domain PS-OCT technology and polarization maintaining fiber optics (Fig. 1(C)). A superluminescent diode (SLD, Superlum, Ireland) centered at *λ* = 837 nm and covering a FWHM bandwidth of 52 nm served as a light source. The measured axial resolution was 7.6 *μ*m in air, corresponding to 5.6 *μ*m in brain tissue (n = 1.35^46^). Point spread functions from an axial resolution measurement are shown in Supplementary Fig. S1(A). The light beam illuminating the sample was circularly polarized. Light backscattered by tissue structures was interfered with the reference beam and detected using a pair of identical homebuilt spectrometers, each featuring a high speed line scan camera (Basler AG, Germany; 2048 of 4096 pixels read out). The PS-OCM system was operated at an A-line rate of 70 kHz.

In the sample arm, a magnifying telescope was used in order to relay the pivot point of a pair of galvanometer scanners onto the back focal plane of a microscope objective. Objectives of different magnifications between 10× and 40× were used to provide high lateral resolution (~4 *μ*m and ~1 *μ*m, respectively). A 10× water immersion objective was used for some samples in order to reduce the strong reflection at the air-tissue interface. Formol or phosphate buffered saline was used for immersion and for preventing the samples from drying. The illuminating beam diameter was chosen to intentionally underfill the objectives’ back apertures such that vignetting effects during x-y scanning were minimal. The effective numerical apertures were between 0.072 for the 10× objective (0.096 with water immersion) and ~0.31 for the 40× objective. Samples were placed in a petri dish located on a three axis manual translation stage (Thorlabs, Germany), which enabled precise alignment of the sample with respect to the beam. By raster scanning the beam over the tissue, PS-OCT data cubes covering a region of up to 1.8 mm (x) × 1.8 mm (y) × 2.7 mm (z, optical distance with n = 1.35) were recorded. Each data set comprised 700 (x) × 700 (y) × 1024 (z) voxels and was acquired in approximately 7.5 seconds.

### PS-OCT image processing

From the acquired spectral data, PS-OCT images including reflectivity, phase retardation and birefringent axis orientation images were computed as described in detail in Appendix A (Supplementary Information)^47^. In addition to cross-sectional B-scan images, 3D renderings as well as projection images (maps, cf. Fig. 1(D)) along depth (z) were generated. Birefringence maps were computed for vessel data by fitting linear slopes to the phase retardation data along depth. Average vessel wall birefringence was computed by first inscribing and circumscribing circles to the birefringence maps and then computing histograms of the birefringence distribution in the vessel wall between the two circles.

### Computational plaque model

**Figure 2:**
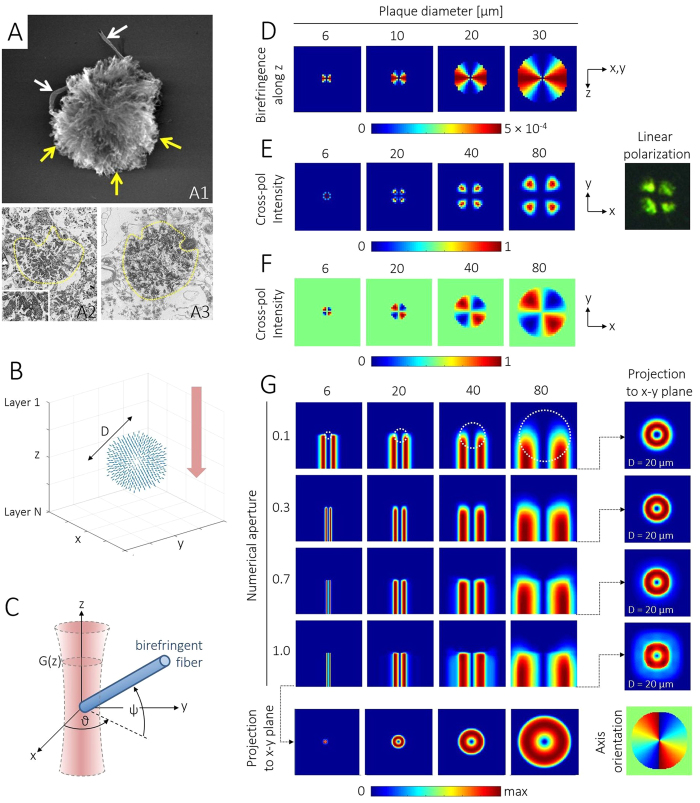
Simulation of PS-OCM imaging of a neuritic plaque. (**A**) Electron micrographs show star-shaped arrangement of A*β* strands emerging from the core. The 3D electron micrograph in (A1) was reproduced with permission (Creative Commons CC-BY license, http://creativecommons.org/licenses/by/4.0/) from G. Plascencia-Villa *et al*., Sci. Rep. 6, 24873 (2016)^48^. (A2-3) Transmission micrographs. (**B**) Computational model of plaque with A*β* fiber orientations shown in blue. Illumination from top to bottom along z-direction (red arrow). (**C**) A Gaussian beam is focused into the plane *z* = 0. For every spatial coordinate, the polarization signal is evaluated within the cross-section *G(z*) of the beam propagating along *z*. An exemplary birefringent fiber with an optic axis orientation *θ* and an inclination angle *ψ* is shown in blue color. (**D**) Calculation of apparent birefringence acting on a beam in *z*-direction penetrating plaques of various sizes. The highest values can be observed where the birefringent fibers are orthogonal to the beam. These data served as input for the following optical ray propagation simulation. (**E**) Simulation of cross-polarized intensity patterns observed for plaques transilluminated with linearly polarized light. This geometry corresponds to the Maltese cross patterns seen in classical polarized light microscopy, see plaque on the right (adapted from L.-W. Jin *et al*., Proc. Nat. Acad. Sci. 100, 15294–15298 (2003)^22^. Copyright (2003) National Academy of Sciences, USA). (**F**) Intensity pattern for illumination with circularly polarized light and an analyzer orientation of 0°. (**G**) Matrix of simulated cross-sectional PS-OCM retardation images for different illumination NA’s and plaque diameters, respectively. The plaque outlines are indicated by white dashed lines in the top row. Within the plaques, a cumulative retardation increase can be observed as a trend towards warmer colors. The retardation projected to the bottom x-y plane of the simulated volume is shown for different NA’s and plaque diameters at the right and in the bottom line, respectively. In the lower right corner, the simulated axis orientation pattern for an 80-*μ*m plaque is shown (color map range: −90° to +90°).

The 3D propagation of a focused polarized light beam through a neuritic A*β* plaque was simulated. The computational steps of this simulation are just briefly described here and discussed in full detail in Appendix B (Supplementary Information). Based on the literature^22^,^48^ and our electron microscopy data (Fig. 2(A)), neuritic plaques were modelled as a radially oriented distribution of birefringent fibers illuminated by a light beam as shown in Fig. 2(B). The tissue polarization effects along beam direction were computed based on Jones calculus^49^. In this formalism, the electric field is described as a two-element vector with a horizontally and a vertically polarized component. The transition of an optical element by the light beam can then simply be computed by multiplying the vector by the according Jones matrix representing the polarization characteristics. In case of the neuritic plaques modelled here, every voxel in the volume was assigned a corresponding Jones matrix. The cortical tissue surrounding the birefringent A*β* sphere was assumed to preserve polarization^43^ (Fig. 2(D)).

Three different settings were analyzed. First, a linearly polarized light beam was propagated once through the volume including a birefringent plaque and the co- and cross-polarized components were computed. This setting resembles classical polarized light microscopy (see sketch in Supplementary Fig. S2(A)) and aids our understanding of the usually cross-shaped appearance of neuritic plaques. Second, a circularly polarized light beam was chosen to illuminate the volume (single pass). Third, a circularly polarized light beam was modelled similar to the sample beam in PS-OCT and computationally backscattered by the plaque and grey matter tissue (double pass), such that virtual PS-OCT images could be calculated. Volumetric simulations were performed for plaque diameters of 6 *μ*m, 20 *μ*m, 40 *μ*m and 80 *μ*m, since the size of neuritic plaques in AD patients varies from 2 to 200 *μ*m^50^, and for illumination beam numerical apertures (NA) of 0.1, 0.3, 0.7 and 1.0, respectively.

## Results

### Simulation of plaque birefringence images

Figure 2(E–G) present simulation results of polarization-contrast images of birefringent A*β* plaques. The effect of cross-polarized detection after transillumination with linearly polarized light is highlighted in Fig. 2(E). Here, signals will only be detected if the sample is birefringent and if the birefringent fiber orientation aligns neither with the illuminating polarization plane (*θ* = 90° in Fig. 2(C)) nor with the analyzer orientation (*θ* = 0°). Hence, the majority of the image areas in Fig. 2(E) appears in blue owing to a lack of cross-polarized signal. Only at locations where the radially oriented fibers form a nonzero angle with both x- and y-axis, a signal can be observed. These signals piece together to the Maltese cross patterns seen at neuritic plaque locations in histologic sections investigated by classical polarized light microscopy^22^.

In order to study the effect of circularly polarized light on the observed polarization component for an analyzer orientation of *θ* = 0°, the second set of simulations was performed. Again, plaques sized 6–80 *μ*m were simulated. The results are shown in Fig. 2(F). Since the quarter wave plate in the illumination path transforms linearly polarized into circularly polarized light, a non-zero signal can be observed even in grey-matter-only regions as well as within the plaque. However, the positive and negative retardation induced to the probe light by the birefringent fibers oriented around *θ* = ±45^°^ cause an increase (red) or decrease (blue) of the signal in the respective areas after passing the analyzer. As a result, the intensity pattern is subject to an azimuthal variation. To validate these simulation results, we performed polarized light microscopy of potato starch samples, which have a radial, birefringent substructure similar to that of neuritic plaques shown in Fig. 2(B). The results of these transillumination experiments performed with linearly and circularly polarized light, respectively, are described in the Supplementary Information (Supplementary Fig. S2) and were in good agreement with the simulations shown in Fig. 2(E,F).

PS-OCT signals arising from birefringent A*β* plaques were computed in the third set of simulations. A matrix of cumulative phase retardation images for simulated plaque diameters of 6–80 *μ*m and for NA’s of 0.1–1.0 is shown in Fig. 2(G). Here, cross-sectional images in the x-z plane are displayed analogous to OCT B-scan images, where the beam propagates from the top towards the bottom of the images before the light is backscattered and returning to the top again.

No phase retardation change was observed in most of the grey matter tissue surrounding the plaques. Within the plaques, which are demarked by a dotted white circle in the top row, a cumulative increase of retardation visible as a color change from blue to red from top to bottom was caused by the birefringent fibers. Beneath the plaques, the retardation values stay at the plaques’ maximum value. Note that all images are scaled from zero to maximum retardation of the respective image in order to enable a direct comparison between the different scenarios. The focal plane was set to the central z-plane of the volume which reflects in the tightest focus at high NA. For low NA values, the plaques cast straight retardation shadows in z-direction. For higher NA values, ray components impinging at greater angles *ψ* contribute to a lateral spread of these retardation shadows (lower rows). This effect is very pronounced in the x-y projections shown for a 20-*μ*m plaque at the right-hand side.

### Three-dimensional PS-OCM imaging of neuritic A*β* plaques

**Figure 3:**
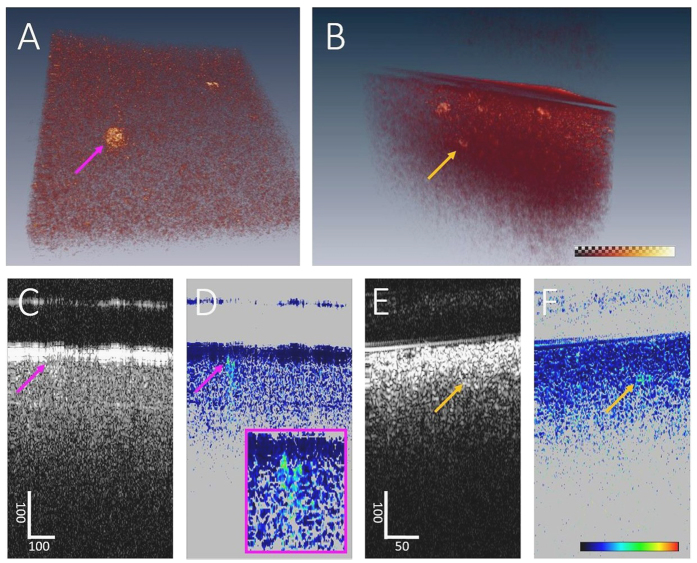
PS-OCM imaging of neuritic plaques in AD brains. (**A**) Volume rendering showing increased phase retardation of A*β* plaques in the cortex of the frontal lobe. Dataset dimensions: 600 *μ*m (x) × 600 *μ*m (y) × 400 *μ*m (z). (**B**) Volume rendering of cortical detail of the hippocampal formation. Dataset dimensions: 360 *μ*m (x) × 360 *μ*m (y) × 400 *μ*m (z). The color maps are scaled from 7° to 41° with reduced transparency at higher retardation values (cf. checkerboard visibility). (**C**) Reflectivity B-scan image sectioning the plaque indicated by the pink arrow in (**A**). (**D**) Phase retardation data of the same B-scan. In contrast to the reflectivity image, the plaque stands out by increased phase retardation. Background pixels with low reflectivity are masked in grey. (**E**) Reflectivity B-scan sectioning the plaque indicated by the yellow arrow in (**B**). (**F**) Corresponding phase retardation showing the A*β* plaque located approximately 100 *μ*m below the sample surface. Scale bars are in micrometers.

Cortical tissue of AD patients was imaged with the PS-OCM system. Exemplary 3D phase retardation images are shown in Fig. 3. In the renderings of samples from the frontal lobe and the hippocampal formation, the polarization-preserving cortex exhibits low retardation values (Fig. 3(A) and (B)). Birefringent plaques can be observed at locations of elevated phase retardation. Cross-sectional images of the data from the frontal cortex are shown in Fig. 3(C–F). The reflectivity images are marked by strong specular reflections at the air-tissue interface and fairly homogeneous reflectivity within the tissue (Fig. 3(C,E)). In the corresponding phase retardation images, the cortical tissue also appears homogeneous with low retardation values, except for the location of the plaque indicated by the pink and yellow arrows where phase retardation is increased (Fig. 3(D,F)). Beneath the plaque in Fig. 3(D), a tail of decreasing retardation values can be observed. Note, however, that such tails are not visible beneath all plaques, see for instance the one in Fig. 3(F) which is located close to the penetration limit of the sample light.

**Figure 4:**
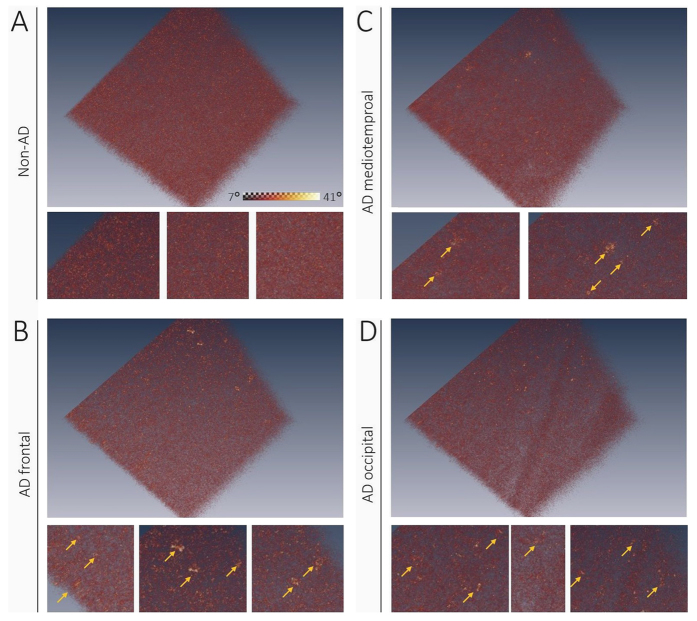
Neuritic plaques in different brain regions. (**A**) Non-AD subject does not show any birefringent plaques in phase retardation image of the frontal cortex. (**B**–**D**) In the cortex of an AD brain, neuritic plaques light up with increasing values in retardation image. Inserts below each rendering show details where plaques are marked by yellow arrows. Color map in (**A**) applies to all images.

Larger phase retardation renderings of three representative regions in an AD brain are shown in Fig. 4 along with a dataset of a non-AD subject. The cortex of the non-AD brain appears uniformly polarization-preserving. A*β* plaques with increased phase retardation were observed in cortical tissue of the hippocampal formation, the frontal lobe and the occipital lobe of the AD patient. Inserts below the renderings show details of some plaque-laden regions.

**Figure 5:**
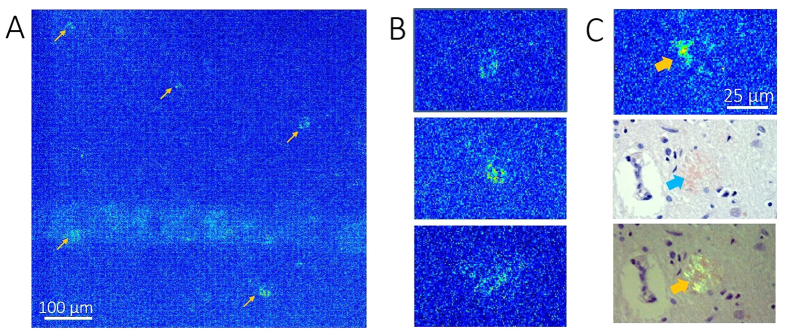
A*β* plaques imaged by PS-OCM and Congo red stained by microscopy. (**A**) Maximum phase retardation projection image. Increased phase retardation can be observed at the locations of A*β* plaques. The bluish streak across the lower part of the image is a retardation artifact caused by a strong backreflection from the formol film covering the sample. (**B**) Details of (**A**) show the signals of individual plaques. (**C**) Comparison of neuritic plaque appearance in retardation image (top), bright field microscopy of Congo red stained section (middle), and polarization contrast microscopy of Congo red stained section (bottom). Under Congo red stain, the plaque appears red in bright field and “apple green” in polarization contrast microscopy.

PS-OCM was also performed in thin sections of cerebral tissue. Figure 5 shows maximum phase retardation projections of a cortical scan. Zoom-ins on four exemplary plaques exhibiting increased retardation are displayed in Fig. 5(B). A comparison of a retardation image and representative micrographs of a Congo red stained A*β* plaque located at an adjacent location in the same sample is shown in Fig. 5(C).

### PS-OCM of vessel walls in CAA

In order to investigate whether PS-OCM can detect increased birefringence in the vessel walls related to amyloidosis, selected cortical and meningeal vessels were imaged in 3 brains diagnosed with CAA and 2 control brains. For this purpose, larger vessels were identified in temporal, frontal and occipital tissue samples, respectively. PS-OCM imaging was performed with the beam oriented roughly perpendicular to the vessel cross-section, i.e., parallel to the vessel direction. Twenty-one 3D data sets in CAA brains and seven control data sets were acquired, each covering a field of view of 1.8 mm (x) × 1.8 mm (y) and containing at least one vessel.

**Figure 6:**
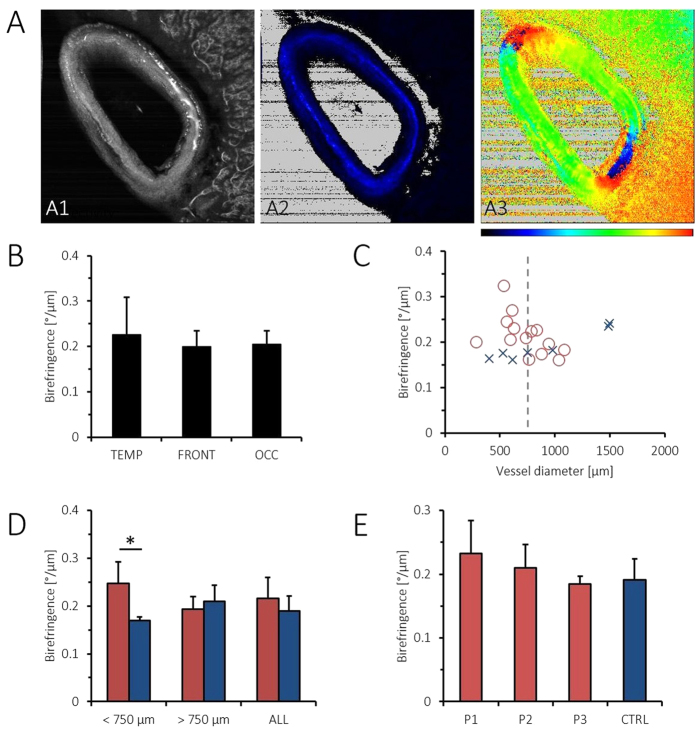
PS-OCM imaging of cortical and meningeal vessel walls in CAA. (**A**) PS-OCM z-projection images (1.8 mm × 1.8 mm). The reflectivity image (A1) shows backscatter contrast of a vessel in vicinity of the cortex. Birefringence in the vessel wall can be observed in the retardation image (A2). The varying orientation of birefringent fibers in the vessel wall is well visible in the axis orientation image (A3). Color maps range from 0° to 90° in (A2) and from −90° to +90° in (A3). (B) Pooled data of vessel wall birefringence in the temporal (TEMP), frontal (FRONT) and occipital lobe (OCC), respectively, do not show significant differences. (**C**) Plot of average vessel wall birefringence vs. vessel diameter. Data from CAA brains is shown as red circles, while control data (CTRL) is shown as blue crosses. (**D**) Birefringence of vessel walls in CAA brains vs. controls. The statistically significant difference of p < 0.01 is indicated by an asterisk. (**E**) Birefringence of vessel walls in individual CAA patients vs. controls.

PS-OCM projection images were computed (Fig. 6(A)). Using the reflectivity z-projection images, the respective maximum (*d*_*max*_) and minimum vessel diameters (*d*_*min*_) were measured. The average vessel diameter was computed as 
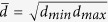
 and amounted to 768 ± 311 *μ*m (mean ± standard deviation; range: 282 *μ*m–1498 *μ*m). Average vessel wall birefringence was 0.21 ± 0.04 °/*μ*m (range: 0.16 °/*μ*m–0.32 °/*μ*m). In Fig. 6(B), vessel wall birefringence is plotted for the pooled data of the temporal, frontal and occipital lobes, respectively. Similar birefringence characteristics were observed in all regions. Mean birefringence was plotted against vessel diameter (Fig. 6(C)). In the pool of smaller vessels from CAA brains (<750 *μ*m, *n*_*CAA*_ = 11), birefringence was significantly higher than in the control vessels (*n*_*CTRL*_ = 4, Mann-Whitney U-test, p < 0.01). In contrast, in larger vessels, (>750 *μ*m, *n*_*CAA*_ = 10, *n*_*CTRL*_ = 3), no significant difference was observed. When comparing vessel wall birefringence in all CAA versus non-CAA brains, a trend (yet no statistical significance) of slightly higher values (0.22 ± 0.04 °/*μ*m) in CAA than in controls (0.19 ± 0.03 °/*μ*m) was observed, Fig. 6(D). Vessel wall birefringence did not show significant differences for any of the three CAA patients versus control data when vessels of all sizes were pooled for the respective subjects (Fig. 6(E)).

## Discussion

Cerebral A*β* plaques are a major hallmark of AD. A variety of imaging methods have been developed for the detection of cortical A*β*, including PET^13^,^50^,^51^, MRI^52^,^53^, and optical imaging^54^,^55^,^56^. OCT in AD patients was, thus far, mostly applied for investigating structural retinal changes such as nerve fiber layer thinning related to neuronal loss^57^,^58^,^59^. Preclinical OCT studies have investigated cerebral perfusion and amyloidosis in mouse models of AD^36^,^60^. In this article, we translated the birefringence-based assessment of A*β* plaques used in histopathological practice to PS-OCT imaging. We demonstrate that individual plaques can be identified and portrayed three-dimensionally with micrometer-scale resolution (Figs 3, 4 and 5). In contrast to other imaging modalities, no exogenous agents are required since the inherent polarization properties of the neuritic plaques are sufficient. Based on literature and electron microscopy data, birefringence images of neuritic A*β* plaques were simulated. The plaque model images nicely reproduced the familiar appearance of real-world tissue observed in polarization contrast microscopy (Fig. 2(E) and Supplementary Fig. S2) and, at least to a certain extent, in the PS-OCM phase retardation data. As such, for instance the plaque in Fig. 3(D) compares well with the simulations in Fig. 2(G). Here, PS-OCM visualizes neuritic plaques exhibiting birefringence owing to systematically oriented fibers and corresponds well with Congo red staining, whereas no diffuse plaques, which have a more random orientation but could be traced by immunohistochemical stains, were detected. In neuritic plaques, the cumulative single-pass retardation to be expected will be on the order of ~1.7° per every 10 *μ*m of plaque thickness. Hence, at the wavelength of 840 nm used here, only larger plaques causing a substantial retardation will be detectable by PS-OCM. Since the precision of phase retardation measurements depends on the signal intensity, a high signal-to-noise (SNR) is required to detect small A*β* plaques inducing only modest retardation. An analysis of the dependence of retardation measurement precision on the SNR of the PS-OCM imaging setting used here is provided in Supplementary Fig. S1(B). For the typical SNR of 19.6 dB observed in cortical brain samples shown in Fig. 3(C), the precision of phase retardation measurements is ~3.5°. Using the above estimate for retardation caused by A*β* plaques, this precision translates to a minimum detectable plaque size of ~20 *μ*m for the PS-OCM imaging setting employed here. The detection capabilities of PS-OCM for small A*β* plaques may be further improved by image averaging^45^,^61^, advanced detection schemes as in dark-field OCM^36^, or the use of broadband light sources providing higher axial resolution and smaller speckle size^62^. Alternative ways of visualizing the confined, localized polarization changes caused by the plaques could be the application of metrics such as the degree of polarization uniformity (DOPU)^63^ or local birefringence evaluation^64^.

There are some limitations to the current imaging approach. First, in order to increase the currently rather limited 3D coverage of our PS-OCM, the technique could be implemented at longer wavelengths, which would enable deeper penetration into cerebral tissue^65^,^66^. By serial scanning, as was demonstrated using OCT imaging with a vibratome and/or an x-y translation stage, large tissue coverage could be achieved by mosaicking of OCT volumes^44^,^67^. Densely sampled PS-OCM volumes could then enable 3D measurements of cerebral A*β* plaque load. Still, OCT is a rapid imaging modality and datasets of several gigabytes of data can be acquired in just a few seconds. Second, it is known that formalin fixation is accompanied by both structural changes such as tissue shrinkage and changes of optical properties^68^,^69^. Both the refractive index and birefringence are impacted by the fixation process and could therefore lead to a false estimation of A*β* plaque load in PS-OCM volumes^69^. Third, while the cerebral cortex surrounding the plaques did not affect the polarization state of light, its neighborhood includes a number of birefringent structures such as white matter tracts as well as collagen fibers in the dura mater and in the walls of larger cerebral vessels. Therefore, care has to be taken to confirm the image location prior to interpreting birefringence images – not every pixel with increased phase retardation is necessarily an A*β* plaque. Finally, the image data only included patients with advanced AD. In future studies, it will be interesting to investigate tissue samples of patients at earlier stages in order to get an impression of the diagnostic sensitivity of PS-OCT during disease progression. An exciting application of the technique presented here would be diagnostic imaging of the retina in AD patients where disease-related neuropathologic features – including A*β* plaque deposition – were also reported^70^,^71^,^72^. In preclinical experiments investigating A*β* plaque load in a mouse model of AD, *in vivo* imaging of retinal plaques was demonstrated based on fluorescent labeling by systematic curcumin administration^71^,^73^. Since retinal A*β* plaques were detectable even earlier than in the brain and accumulated with disease progression in the transgenic mice, retinal scans revealing A*β* load may serve as an easily accessible target for tracking disease progression also in human patients^71^. In light of these findings, PS-OCT could be used for spotting lesions or even for early diagnosis of AD by scanning the retina, however with the advantage of being both a relatively cheap, rapid and noninvasive technique for 3D imaging but without the need for exogenous contrast agents, as A*β* plaques provide intrinsic polarization contrast.

The vasculature of the CNS is affected by amyloidosis in CAA^74^. We hypothesized that amyloid deposits would alter the birefringent properties of the vessel wall and used PS-OCM to investigate the birefringence of cortical and meningeal vessel walls in brains affected by CAA (Fig. 6). Due to the fibrous nature of vessel walls, also healthy vessels are birefringent. This birefringence, which was also exploited as a contrast channel in intravascular PS-OCT implementations^75^,^76^, adds up with the birefringence of amyloid beta deposits. In our pilot experiment including 21 PS-OCM volumes of cortical and meningeal vessels in CAA brains and seven control data sets, a difference of vessel wall birefringence was only found for vessels measuring less than 0.75 mm in diameter. In line with this finding, histologic investigations using Congo red staining have shown that smaller vessels were more strongly affected by amyloidosis than larger ones^74^,^77^. However, since the number of subjects (5) and specimens (28) was limited in this pilot experiment using PS-OCT, a more extensive study is required in order to provide strong statistical evidence. Also, arteries were reported to be more frequently affected by A*β* deposition than veins^77^, a factor we did not investigate here. Finally, in order to rule out birefringence alterations by formalin fixation, PS-OCT of native material or *in vivo* in animal models of CAA would be an interesting continuation of the experimental results presented here.

## Conclusion

In this paper, we have demonstrated PS-OCM imaging in unstained, formalin-fixed brain samples of human AD and CAA patients. Based on their intrinsic birefringence properties, neuritic plaques were successfully visualized in representative cortical regions. In line with the results from PS-OCM imaging, the three-dimensional structure of senile A*β* plaques was computationally modelled for different illumination settings and plaque sizes. Furthermore, the birefringent properties of cortical and meningeal vessel walls in CAA were investigated. Significant differences were found in smaller vessels; however, the sample size for this experiment was small. In summary, PS-OCT may be a useful tool for three-dimensional imaging of amyloidosis based on intrinsic polarization properties.

## Additional Information

**How to cite this article:** Baumann, B. *et al*. Visualization of neuritic plaques in Alzheimer’s disease by polarization-sensitive optical coherence microscopy. *Sci. Rep.* **7**, 43477; doi: 10.1038/srep43477 (2017).

**Publisher's note:** Springer Nature remains neutral with regard to jurisdictional claims in published maps and institutional affiliations.

## Supplementary Material

Supplementary Information
