# Supplementary material for: Visualization of neuritic plaques in Alzheimer’s disease by polarization-sensitive optical coherence microscopy
Source: Sci Rep. 2017 Mar 6;7:43477. doi: 10.1038/srep43477 (PMC5337955; doi:10.1038/srep43477)
Supplement: Supplementary Information [file srep43477-s1.pdf]

# Visualization of neuritic plaques in Alzheimer’s disease by polarization-sensitive optical coherence microscopy

Bernhard Baumann[1], Adelheid Woehrer[2], Gerda Ricken[2], Marco Augustin[1], Christian Mitter[2,3], Michael Pircher[1], Gabor G. Kovacs[2], Christoph K. Hitzenberger[1]

[1]Medical University of Vienna, Center for Medical Physics and Biomedical Engineering, A-1090 Vienna, Austria
[2]General Hospital and Medical University of Vienna, Institute of Neurology, A-1090 Vienna, Austria
[3]General Hospital and Medical University of Vienna, Department of Radiology, A-1090 Vienna, Austria

# Supplementary information

## Appendix A - Computation of PS-OCT images

In a spectral domain PS-OCT system, the polarization state of light backscattered by the sample and light interfered with a reference beam is analyzed[1]. For this purpose, the light beam at the interferometer exit is split into a horizontally ($H$) and a vertically ($V$) polarized beam component (Fig. 1(C)). Each of these components is detected by a spectrometer which disperses the broadband light and records the interference signal as a function of wavelength. From the spectral data of the two polarization channels, axial depth scans (A-scans) can be computed using the Fourier transform[2]. The resulting complex-valued signals $A_H \exp[i\Phi_H]$ and $A_V \exp[i\Phi_V]$ now provide the basis for computing PS-OCT signals – reflectivity $R(z)$, phase retardation $\delta(z)$ and fast optic axis orientation $\theta(z)$ – in the following manner[3]:

$$R(z) \propto A_H^2 + A_V^2 \tag{1}$$

$$\delta(z) = \arctan(A_V/A_H) \tag{2}$$

$$\theta(z) = \frac{1}{2}(\Phi_V - \Phi_H + \pi) \tag{3}$$

where $H$ and $V$ denote the horizontal and the vertical polarization channel, respectively. The measurement ranges are [0 – $\pi$/2] and [-$\pi$/2 – $\pi$/2] for retardation and axis orientation values, respectively. When the image data is displayed using a color map, rainbow color maps ranging from blue (-90°) to red (+90°) were used for the axis orientation images. For the phase retardation data, rainbow color maps were used for the 2D images, whereas a black-red-yellow-white color map (termed "hot" in Matlab or Python) was used for the volume renderings in order to improve visibility of the neuritic plaques. In both retardation and axis orientation imagery, areas with low reflectivity $R(z)$ were usually set to grey.

## Appendix B – Computational plaque model

Based on the literature[4,5] and our electron microscopy data (Fig. 2(A)), propagation of a focused polarized light beam through a neuritic A$\beta$ plaque was simulated. A 3D grid of Jones matrix voxels sized 1 $\mu$m × 1 $\mu$m × 1 $\mu$m was defined. In the center of the volume, a plaque with diameter $D$ was modelled as a sphere of symmetrically radiating birefringent fibers (Fig. 2(B)), i.e. by a field of Jones matrices[6]

$$J_{plaque}(r) = T(\theta)\Lambda(\delta)T(-\theta) \tag{4}$$

where $\theta$ and $\psi$ are the birefringent fiber angles in the plane orthogonal to the beam direction $z$ (with $\theta$ = 0 oriented in $+x$ direction) and with respect to the $x$-$y$ plane, respectively (Fig. 2(C)). Spatial coordinates within the volume are represented as $r = (x,y,z)$. $T(\theta)$ and $T(-\theta)$ are rotation matrices, and $\Lambda = \begin{pmatrix} \exp(i\delta/2) & 0 \\ 0 & \exp(-i\delta/2) \end{pmatrix}$ is a linear retarder matrix where $\delta = \frac{2\pi}{\lambda}\Delta n h \cos^2\psi$ is the retardation of a voxel (height $h$) when illuminated at an angle $\pi/2 - \psi$ to the fiber orientation (cf. Fig. 2(C)). (Hence, the observed retardation for a beam illuminating parallel to the fiber, i.e. at $\psi = \pi/2$, would be 0). A birefringence of $\Delta n = 4 \times 10^4$ was assumed for the unstained amyloid fibers[7]. The background surrounding this plaque represented grey matter and was set to be polarization preserving, $J_{GM}(r) = \mathbb{1}$. Exemplary cross-sectional images showing the distribution of retardation per pixel (i.e., the apparent birefringence) for simulated plaques with different diameters $D$ are displayed in Fig. 2(D).

The illuminating light beam was modelled as a focused Gaussian beam with a numerical aperture $NA = \sin(\alpha)$ between 0.1 and 1.0. The beam focus had corresponding waists $w_0 = \lambda/\pi\alpha$ and was set to the central $x$-$y$ plane of the volume ($z$ = 0). For every depth $z$, the respective beam waist $w(z) = w_0\sqrt{1+(z/z_R)^2}$ was calculated, where $z_R = \pi w_0^2/\lambda$ denotes the Rayleigh range. Using $w(z)$, two-dimensional and finally 3D Gaussian beam intensity profiles were computed.

Three sets of simulations with different input polarization states and beam trajectories were performed:

(i) Transillumination with linearly polarized light (corresponding to the standard setting in classical polarization contrast microscopy)

(ii) Transillumination with circularly polarized light (single pass)

(iii) Illumination with circularly polarized light and polarization sensitive detection of scattered light (double pass, corresponding to the standard PS-OCT setting, cf. our approach described in the Methods section)

In these simulations, the polarized light beam was represented by a Jones vector $j_{in}$ which was launched at every $x$-$y$ coordinate at the top of the volume and propagated towards the focus. For the first set of simulations (i), each of the rays was initially in a vertical linear input state, $j_{in} = [0,1]^T$, in order to model light propagation in a polarized light microscope. In order to achieve circularly polarized light for (ii) and (iii), $j_{in}$ was passed through a quarter wave plate oriented at 45° by multiplication with the corresponding Jones matrix ($J_{QWP} = \frac{1}{2}\left(\begin{smallmatrix} 1-i & 1+i \\ 1-i & 1+i \end{smallmatrix}\right)$ ), thus $J_{QWP}\, j_{in}$. Now, for every spatial coordinate $r = (x,y,z)$ the apparent phase retardation $\overline{\delta}$ and axis orientation $\overline{\theta}$ seen by the Gaussian beam were computed by spatially averaging the $\delta$ and $\theta$ values within the Gaussian beam cross-section. Thereby, the corresponding Gaussian weight $G(z)$ was applied:

$$\overline{\delta}(r) = |\sum_m G_m \delta_m e^{i2\theta_m}| \tag{5}$$

$$\overline{\theta}(r) = \frac{1}{2} \arg[\sum_m G_m \frac{\delta_m}{\sum_l \delta_l} e^{i2\theta_m}] \tag{6}$$

where $m$ represents the pixel indices within the beam waist $w(z)$ and $\sum_m G_m = 1$. The Jones matrix $J_k(r)$ can now be calculated from $\overline{\delta}$ and $\overline{\theta}$ similar to equation 4. For every spatial coordinate $r$, the Jones vectors resulting from the propagation of the respective rays could be iteratively computed by cumulating the Jones matrices along the beam while passing through layers $1-k$,

$$j_k(r) = J_k J_{k-1} \cdots J_1 j_{in}. \tag{7}$$

However, since it turns out that for every lateral position, $\overline{\theta}(x, y = const.)$ remains constant within and beneath the plaque, the computation of $j_k(r)$ can be vastly sped up by simply calculating

$$J_k^{cum}(r) = J(\overline{\theta}; \overline{\delta}_k^{cum} = \sum_{j=1}^{k} \overline{\delta}_j). \tag{8}$$

For simulations (i) and (ii), the co- and cross-polarized components of the field vectors $j_k(r)$ were extracted and the corresponding intensities were calculated as the square of the fields and displayed.

In order to account for the double pass of the polarized light beam through the volume and to simulate the PS-OCT signal in (iii), the cumulative Jones matrices $J_k^{cum}(r)$ as well as $J_{QWP}$ were applied another time:

$$j_{PS}(r) = J_{QWP} J_k^{cum}(r) J_k^{cum}(r)\, j_{in}. \tag{9}$$

where $j_{in}$ denotes the circularly polarized input beam. From the resulting Jones vector components resembling the signal detected by PS-OCT, cumulative phase retardation images were computed for every simulated volume in the usual manner as described in Appendix A[1]. Note that for all simulations, the same optical properties (transmission, attenuation, backscattering) were assumed for plaque and grey matter tissue. Diattenuation was assumed negligible.

The size of neuritic plaques in AD patients varies from 2 to 200 $\mu$m[8]. Hence, volumetric simulations were performed for plaque diameters of 6 $\mu$m, 20 $\mu$m, 40 $\mu$m and 80 $\mu$m, and for illumination beam NA's of 0.1, 0.3, 0.7 and 1.0, respectively.

## Supplementary figures

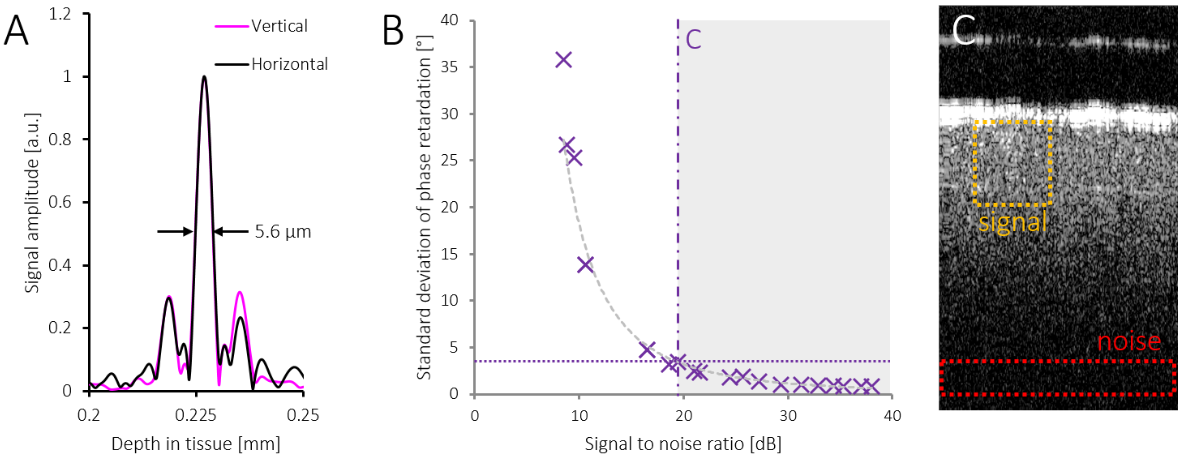


**Figure S1.** Characterization of axial resolution and dependence of the precision of phase retardation measurements on the signal-to-noise ratio (SNR). (A) Point spread functions measured with a mirror as a sample are shown for the vertical and horizontal polarization channels. The axial resolution, defined as the FWHM of these functions, was 5.6 $\mu$m in both channels. (B) Phase retardation measurement precision vs. SNR. The sample arm was fitted with a mirror, a wave plate providing a retardation of 58° at 840 nm wavelength (Linos D-$\lambda$/4-1300), and a variable neutral density filter leaving the choice of different SNRs. Sequences of 1000 PS-OCT A-scans were acquired at 21 SNR levels between 8 dB and 38 dB. SNR was computed as $10log_{10}[signal^2/\sigma_{noise}{}^2]$. In order to characterize the measurement precision at each SNR level, the standard deviation $\sigma_{ret}$ of phase retardation measurements at the mirror location was calculated for every sequence. A substantial precision decrease at lower SNR levels can be observed (grey trend line: $\sigma_{ret} = 5891.1x^{-2.511}$). (C) An SNR of 19.6 dB was computed from the mean signal in the yellow box and the standard deviation of the noise floor in the red box shown in the brain scan of Fig. 3(C). The dashed violet line in (B) indicates the corresponding $\sigma_{ret}$ of $\sim$3.5°. The SNR range exceeding 19.6 dB (dash-dotted line) is shaded in grey in (B).

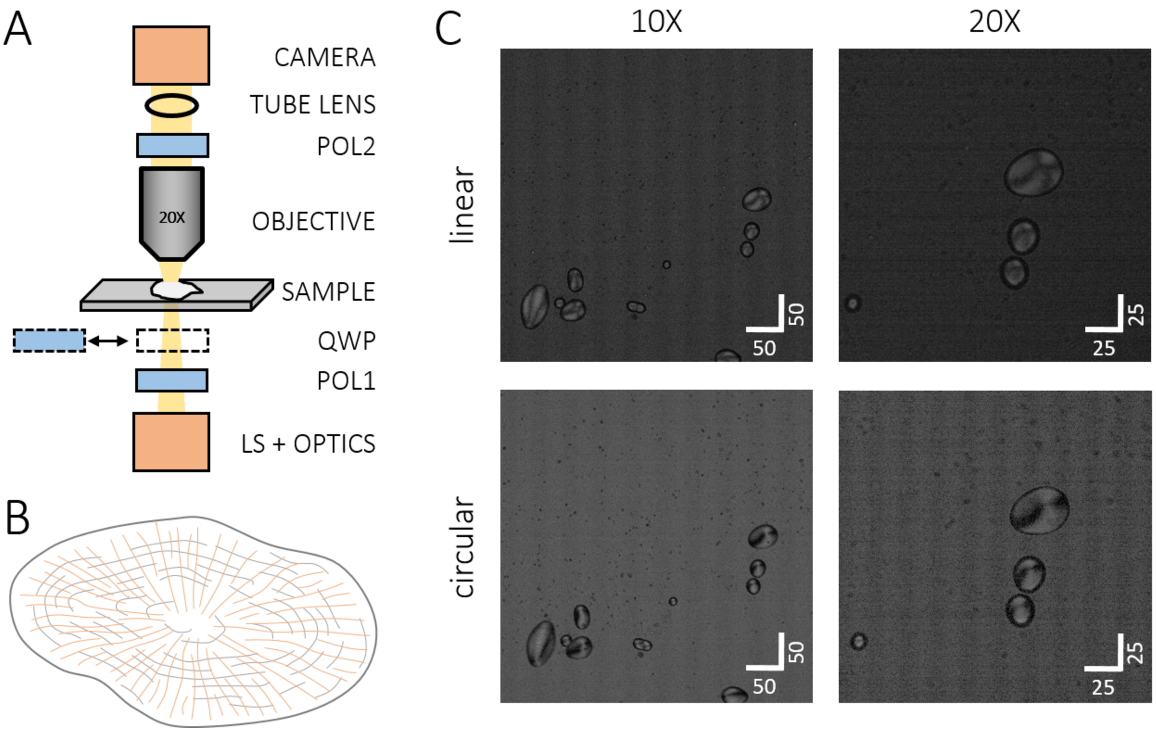


**Figure S2.** Polarized light microscopy with linearly and circularly polarized incident light. (A) Sketch of the optical pathway in the polarized light microscope (Olympus BX51-P). Light from the light source (LS) passed a polarizer (POL1) before illuminating the sample which is imaged onto a CCD camera via an objective, another polarizer (POL2) oriented at 90° with respect to the first, and a tube lens. A quarter wave plate (QWP) oriented at 45° was inserted between the first polarizer and the sample in order to change from linearly to circularly polarized illumination. Potato starch granules (Stärkina, Austria) suspended in phosphate buffered saline were used as a birefringent sample. (B) Cartoon of a starch granule. The crystalline structure of starch granules relies on radially oriented amylopectin molecules indicated by orange lines.[9] (C) Polarized light microscopy of potato starch granules. The sample was imaged using linear (top row) and circularly polarized incident light (bottom row). In case of linearly polarized light, the background appears dark and pronounced, hyperintense Maltese cross patterns can be observed at the starch granule locations. In contrary, under circularly polarized light, each two opposing quadrants of the granules appear hyperintense whereas the other two appear hypointense compared to the background. Images at 10× and 20× magnification are shown in the left and right column, respectively. Scale bars are in micrometers.

## References


**1.** Hitzenberger, C., Gotzinger, E., Sticker, M., Pircher, M. & Fercher, A. Measurement and imaging of birefringence and optic axis orientation by phase resolved polarization sensitive optical coherence tomography. *Opt. Express* **9**, 780–790 (2001).

**2.** Fercher, A. F., Hitzenberger, C. K., Kamp, G. & Elzaiat, S. Y. Measurement of intraocular distances by backscattering spectral interferometry. *Opt. Commun.* **117**, 43–48 (1995).

**3.** Götzinger, E., Pircher, M. & Hitzenberger, C. K. High speed spectral domain polarization sensitive optical coherence tomography of the human retina. *Opt. Express* **13**, 10217–10229 (2005).

**4.** Jin, L.-W. *et al.* Imaging linear birefringence and dichroism in cerebral amyloid pathologies. *Proc. Natl. Acad. Sci.* **100**, 15294–15298 (2003).

**5.** Plascencia-Villa, G. *et al.* High-resolution analytical imaging and electron holography of magnetite particles in amyloid cores of alzheimer's disease. *Sci. Rep.* **6**, 24873 (2016).

**6.** Jones, R. C. A new calculus for the treatment of optical systems i. description and discussion of the calculus. *J. Opt. Soc. Am.* **31**, 488–493 (1941).

**7.** Taylor, D. L., Allen, R. D. & Benditt, E. P. Determination of the polarization optical properties of the amyloid-congo red complex by phase modulation microspectrophotometry. *J. Histochem. Cytochem.* **22**, 1105–1112 (1974).

**8.** Kozlowski, H., Brown, D. R. & Valensin, G. *Metal Complexes in the Brain Imaging and Diagnosis*. Metallochemistry of Neurodegeneration: Biological, Chemical, and Genetic Aspects (Royal Society of Chemistry, Cambridge, UK, 2006), 1 edn.

**9.** Coultate, T. P. *Food: the chemistry of its components* (Royal Society of Chemistry, 2009).
